# Regional Differences in Metabolic Risk in the Elderly in Korea

**DOI:** 10.3390/ijerph191811675

**Published:** 2022-09-16

**Authors:** Ji-Myung Kim, Yun-Jung Bae

**Affiliations:** 1 Food and Nutrition Major, Division of Food Science & Culinary Arts, Shinhan University, Uijeongbu 11644, Korea; 2 Major in Food and Nutrition, Division of Food Science and Biotechnology, Korea National University of Transportation, Jeungpyeong 27909, Korea

**Keywords:** urban, rural, metabolic risk, elderly, Korea

## Abstract

Lifestyle and dietary differences in urban and rural environments have different impacts on elderly health. We aimed to evaluate the nutritional intakes and metabolic risks in the urban and rural elderly. We analyzed 3018 elderly individuals (1358 men, 1660 women) who were aged 65 years and older using the Korea National Health and Nutrition Examination Survey data (2013–2016). Anthropometric data, blood pressure, and blood profiles were collected using health examinations. Daily dietary intakes were analyzed by the 24-h dietary recall method. Rural elderly women had significantly higher triglycerides (TG) levels and lower high-density lipoprotein (HDL)-cholesterol levels than urban elderly women (*p* = 0.014, *p* = 0.005). The rural elderly had higher carbohydrate intake and percentage of carbohydrate energy intake and lower intakes of fat, vitamin A, vitamin B_2_, and vitamin C and percentage of fat energy intake than the urban elderly for both men and women (*p* < 0.05). The odds of high TG and low HDL-cholesterol were 1.66 (95% confidence interval [CI] 1.23–2.23) and 1.33 (95% CI 1.01–1.77), respectively, in elderly women living in rural areas compared to their urban counterparts, after adjusting for confounding factors. Therefore, nutritional intervention might be needed to improve the nutritional status of the elderly in rural areas and to manage low HDL-cholesterol in rural women.

## 1. Introduction

Economic growth and higher living standards, along with medical advancements, have led to an extended human lifespan, and this is a global trend. However, the increase in healthy life expectancy has not kept pace with this increase in life expectancy [1], and, therefore, strategies aimed at improving healthy life expectancy in the elderly have received increasingly more attention. In old age, physical limitations and functional decline appear, which may be accompanied by reduced appetite and loss of interest in daily activities, due to the aging process and chronic diseases; thus, special nutritional management is necessary [2].

Nutritional status is mostly influenced by individual and environmental factors. Individual factors include biological characteristics (e.g., sex, age, genetics), lifestyle (e.g., dietary behaviors, physical activities), and socio-economic conditions (e.g., education level, occupation), while environmental factors include national context (e.g., government nutrition policy, nutrition monitoring system) and community conditions (e.g., food environment, health and nutrition services) [3]. Particularly from a regional perspective, health and nutritional management in rural areas may be more difficult than in urban areas due to low food availability, limited food accessibility, and restricted access to healthcare facilities and medical services. In Korean adults aged 40–64 years, rural residents showed a lower intake of most nutrients, whereas a higher carbohydrate intake and a higher percentage of energy from carbohydrates compared to urban residents [4]. In an earlier study of the elderly, the intake of fruit, milk, and dairy products, which are associated with the intake of vitamin C, vitamin A, carotene, and niacin, was significantly lower among rural elderly than among urban elderly [5]. Moreover, the intake of calcium and potassium is significantly lower in rural elderly compared to urban elderly and the major food sources of calcium and potassium were different in urban elderly and rural elderly people [6]. Given this finding, the possibility is raised that nutrient intake of the elderly in rural areas is generally inadequate compared to urban elderly.

Urbanization leads to a better supply of foods rich in energy and an easier transition to a western-style diet, while rural residents tend to maintain a more traditional diet [7]. Such differences in nutritional intake may be closely related to the onset of chronic diseases. A study of Korean adults aged 40–64 years reported that the odds ratios for metabolic syndrome were significantly higher in rural residents compared to urban residents [4]. In a study of Portuguese adults aged 30 years and older, after adjusting for sex and age, residents in non-urban areas had a significantly higher prevalence of metabolic syndrome [8]. An earlier report showed that the intake of energy and carbohydrate was considerably higher among rural elderly women than among urban elderly women despite no significant differences in the percentage of energy intake from fat between urban and rural areas; moreover, in terms of abdominal obesity and hypertension, there was a significant difference in urban–rural relative risk [9]. Additionally, a recent study found that there was a significant positive correlation between saturated fatty acid intake and higher blood total cholesterol among the elderly in urban areas, but not in rural areas [10]. These regional differences in the prevalence of metabolic syndrome can be explained by many demographic and socio-economic factors [11]. Despite the differences in nutrient intake between urban and rural areas and their substantial impact on metabolic indicators, studies of urban–rural differences in metabolic indicators in relation to nutrient intake are still lacking.

Data from previous studies have identified that regional differences between urban and rural areas affect nutrient intake patterns and changes in metabolic indicators, such as metabolic syndrome. The Korean population is rapidly aging; however, no study has examined the relationship between urban–rural nutrient intake and metabolic indicators in Korean elderly who are at high risk of malnutrition and unbalanced nutrition. Therefore, this study aimed to analyze the risk of urban–rural metabolic indicators in the elderly population in Korea, which is experiencing rapid aging, and to analyze the differences in the relationship between nutrient intake status in urban and rural areas and metabolic indicators. 

## 2. Methods

### 2.1. Study Design and Participants

This study was conducted based on the data of 31,098 people from the KNHANES VI (2013–2015) and VII-1 (2016) [12]. The raw data of a health and behavior interview, a health examination, and a dietary survey, which consisted of KNHAENS, were used. Participants were randomly selected using a multi-step clustering and stratified random sampling methods with proportional assignment based on geographic area, gender, and age group from National Census Data. [13]. Among them, the data of the following subjects were excluded from the analysis of this study. (1) Participants who did not respond to the dietary survey (*n* = 3385); (2) participants under 65 years of age (*n* = 22,233); (3) participants reporting inadequate daily energy intake (<800 or >4000 kcal/day for men and <500 or >3500 kcal/day for women) (*n* = 188) [14]; (4) participants without data on metabolic parameters (*n* = 2099); and (5) participants without socioeconomic characteristics (*n* = 175). A total of 3018 participants (1358 males, 1660 females) were analyzed in the present study. This study was approved by the Institutional Review Board (IRB) of the Korea Centers for Disease Control and Prevention (KCDC) (IRB no: 2013-07CON-03-4C, 2013-12 EXP-03-5C) for the years 2013 and 2014. In the case of the 2015 and 2016 KNHANES, the IRB’s deliberation was exempted in accordance with the Bioethics and Safety Act. Written consent was obtained from all study participants.

### 2.2. Data Collection

#### 2.2.1. Socio-Demographic Factors

Socio-demographic information, such as age, gender, education level, household income, marital status and living area, smoking, drinking, and physical activity status was obtained through a health and behavior interview. Living area was classified into urban (-dong districts) or rural areas (-eup/-myeon districts). Household income was classified into low, mid-low, mid-high, or high. Educational level was classified as lower than elementary school, middle school, or higher than high school. Marital status was divided into married or unmarried. Smoking and drinking both were classified as yes and no. Physical activity was also classified as yes or no according to the KNHANES’s guideline based on the KCDC [15,16].

#### 2.2.2. Anthropometrics and Biochemical Variables

Anthropometric measurements of height, weight, and body mass index (BMI) and blood profiles were measured through a health examination. BMI (kg/m^2^) was calculated by dividing the weight (kg) by the square of the height (m^2^). BMI categories were classified as underweight (BMI < 18.5 kg/m^2^), normal weight (≥18.5 kg/m^2^ BMI < 23 kg/m^2^), overweight (≥23 kg/m^2^ BMI < 25 kg/m^2^), or obese (BMI ≥ 25 kg/m^2^), according to the criteria for Asians [17]. Blood profiles, total cholesterol, HDL-cholesterol, triglyceride (TG), and glucose in fasting blood were directly measured using Hitachi Automatic Analyzer 7600 (Hitachi, Tokyo, Japan). LDL-cholesterol was calculated using the Friedewald equation [18].

#### 2.2.3. Metabolic Abnormalities

Metabolic abnormalities were based on the National Cholesterol Education Program Adult Treatment Panel III criteria [19]. Abdominal obesity was defined as a waist circumference of ≥90 cm for men and ≥ 80 cm for women according to the Asia-Pacific population standard [17]. High TG was determined by a fasting blood TG level ≥ 150 mg/dL. Low HDL-cholesterol was determined by a fasting blood HDL-cholesterol level < 40 mg/dL in men and <50 mg/dL in women. High blood glucose was determined by a fasting blood glucose ≥ 100 mg/dL or medication (insulin or oral agents).

#### 2.2.4. Dietary Intake

Dietary intake data in the nutrition survey were collected by trained dietitians using the 24-h recall method to respond to all food intake during the day before the survey. All types and amounts of food and the time and place of each meal were investigated. Tools, such as measuring cups, portion-size booklets, and photos, were used to accurately track intake. The total energy and nutrient intakes for each individual were calculated using the food composition table published by the Rural Development Administration [20]. In addition, the nutrient intake per 1000 kcal of energy intake and the energy intake ratio of the three major nutrients were also calculated.

### 2.3. Statistical Analysis

For statistical analysis, SAS 9.4 software (SAS Inc., Cary, NC, USA) was used. All analyses were considered for the sampling weights and the complex survey design, which consisted of multistage, stratified, and clustered samples. The participants were categorized into two groups depending on the living area: (1) urban group and (2) rural group. The analyses were conducted separately for men and women. Intake of total energy, macronutrients, and micronutrients was expressed as mean ± standard error. Categorical variables, including age, education, household income, marital status, alcohol intake, smoking, BMI, and metabolic abnormalities (abdominal obesity, high TG, high blood glucose, low HDL cholesterol), were expressed as frequencies and percentages and compared between the urban and rural groups using the chi-square test. Blood profiles and intake of nutrients by sex and living area were analyzed using the general linear model. The odds ratios (ORs) and the 95% confidence intervals (CIs) were calculated, using logistic regression analyses. The prevalence of metabolic abnormalities was compared by quartile of carbohydrate consumption and percentage of energy from carbohydrates according to sex and living area, using multiple logistic regression analyses before (Model 1) and after adjusting variables (Model 2). The adjusted variables included age, household income, education level, physical activity, and total energy intake for men, and age, household income, education level, BMI categories, and total energy intake for women. *p*-values < 0.05 were considered significant.

## 3. Results

The general characteristics of the participants are shown in Table 1. The mean age was significantly higher in the rural group than in the urban group (*p* = 0.049 in men; *p* = 0.010 in women), and the proportion of patients over 75 years old was significantly higher in the rural group than in the urban group in women (*p* = 0.008), but not men. The rural groups had significantly lower rates of education and lower household incomes (*p* < 0.0001 for both) compared to the urban groups. The proportion of physical activity was significantly lower in the rural group than in the urban group in men (*p =* 0.040), but not women. The proportion of underweight was higher in the rural group than urban group in women (*p* = 0.043), but not men.

Table 2 shows the status of the metabolic abnormality indicators of participants. Blood TG level was significantly higher in the rural group than in the urban group in women (*p* = 0.014), but not men. However, HDL-cholesterol level was significantly lower in the rural group than in the urban group in women (*p* = 0.005), but not men. The rural group had a higher prevalence of high TG (*p* = 0.002) and low HDL-cholesterol (*p* = 0.017) than the urban group in women. However, there were no significant differences of the prevalence of metabolic abnormalities between the urban group and the rural group in men.

The daily nutrient intake of participants is shown in Table 3. There were no significant differences in energy intake between the rural group and the urban group for both men and women. In the distribution of energy intake of macronutrients, rural areas showed significantly higher carbohydrate energy ratios (*p* = 0.001 in men, *p* < 0.0001 in women) and lower fat (*p* < 0.0001 for both) and protein energy ratios (*p* = 0.001 in only women) than urban ones. In addition, the rural group had significantly higher carbohydrate intake (*p* = 0.012 in men, *p* = 0.001 in women) and lower fat intake (*p* = 0.019 in men, *p* < 0.0001 in women) than the urban group. The cholesterol intake of the rural group was significantly lower than that of the urban group (*p* = 0.002) in women, but not among men. The intake of the micronutrients showed significant differences among the urban and rural participants; vitamin A (*p* = 0.037 in men, *p* = 0.048 in women), vitamin B2 (*p* = 0.011 in men; *p* = 0.007 in women), and vitamin C (*p* = 0.029 in men; *p* = 0.001 in women) with rural group consumption being significantly lower than those of the urban group. The calcium intake of the rural group was significantly lower than that of the urban group in men (*p* = 0.031), but not in women.

Table 4 shows the ORs for metabolic abnormalities in rural and urban elderly. The crude OR for metabolic abnormalities were significantly higher in the rural group for high TG (OR = 1.56, 95% CI: 1.17–2.09) and for low HDL-cholesterol (OR = 1.38, 95% CI: 1.06–1.80) compared to the urban group in women. After adjusting for potential confounder variables, the adjusted OR for metabolic abnormality was also significantly higher in the rural group for high TG (OR = 1.66, 95% CI: 1.23–2.23) and for low HDL-cholesterol (OR = 1.33, 95% CI: 1.01–1.77) compared to the urban group in women. However, there was no association with OR for metabolic abnormalities among men.

Table 5 shows the ORs for metabolic abnormalities according to the quartile of carbohydrate consumption by sex. Among men, the crude ORs of high TG in the Q2, Q3, and Q4 group were 0.65 (95% CI: 0.46–0.92), 0.68 (95% CI: 0.47–0.98), and 0.69 (95% CI: 0.48–0.99), respectively, compared to the Q1 group (reference), classified according to the carbohydrate consumption. Moreover, the crude OR of high blood glucose in the Q4 group was 0.71 (95% CI: 0.51–0.98) compared to the Q1 group, and significant associations were observed (*p* for trend = 0.014) in men. After adjusting for potential confounder variables, the adjusted OR for only high TG in the Q2 group were 0.58 (95% CI: 0.39–0.88) compared to the Q1 group. Among women, the adjusted ORs of low HDL-cholesterol in the Q3 group and high blood glucose in the Q2 group, as well as the crude ORs, were 2.42 (95% CI: 1.59–3.68) and 0.69 (95% CI: 0.48–0.98), respectively, compared to the Q1 group. Moreover, significant associations were observed for high TG (*p* for trend = 0.036) and low HDL-cholesterol (*p* for trend = 0.040).

Table 6 and Table 7 show the ORs for metabolic abnormalities according to the percentage of energy from carbohydrate. When the subjects were divided into two groups based on the energy ratio of carbohydrates (65% cut-off value), the crude ORs of low HDL-cholesterol in the >65% energy of carbohydrate group were 1.94 (95% CI: 1.06–3.56) compared to the ≤65% energy of carbohydrate group (reference). However, after adjusting for potential confounder variables, there was no association with OR for metabolic abnormalities and carbohydrate energy consumption by living area and sex.

## 4. Discussion

A key finding of this study was that the ORs of high TG and low HDL-cholesterol were significantly higher in elderly women living in rural areas than in their urban counterparts. In addition, rural elderly men and women both showed a significantly higher percentage of energy from carbohydrate than urban elderly men and women. A sub-analysis on the relationship between carbohydrate intake and metabolic risks in urban–rural settings indicated no significant differences between the two settings.

A variety of factors affect the health of the elderly, including individual characteristics (e.g., demographic characteristics, socio-economic status, lifestyles), neighborhood characteristics (e.g., neighborhood walkability, availability of recreational or sports facilities for elderly, access to public transport), and social networks (e.g., social contacts, social support). Activities of daily living and social contacts may affect the association between neighborhood environment and the health of the elderly, which is attributable to significant neighborhood differences between urban and rural areas. Studies have continued to report differences in lifestyles, dietary life, and health indicators of the elderly due to regional differences, such as urban–rural status [4,9,21,22]. According to previous reports, the elderly living in cities tended to have significantly higher education and income levels than those living in rural areas did [22,23,24], and similar results were observed in this study. Low educational attainment was linked to living in areas with poor access to health-care facilities [25]. Some studies have found that among people with hypertension, those with low education showed low hypertension awareness, such as recognition and diagnosis of the condition, inadequate treatment, and control [26]. People with lower education attainment seemed to have reported higher mortality and rate of coronary artery disease [27]. Together, these studies provide evidence that rural elderly with low education and income are less likely to live in a health-promoting environment, but more likely to be exposed to diseases.

In this study, the proportion of elderly men who reported engaging in physical activity was significantly higher among those living in urban areas than their rural counterparts, whereas elderly women had no major differences in physical activity levels between urban–rural areas. The urban elderly and rural elderly may show different patterns of physical activity as cities generally have better walkability, better access to public transport, parks, and recreational facilities than rural areas. Several environmental factors that promote physical activity of the urban elderly also help them to expand their social networks. Considering that physical activity is an important element that can define lifestyle in urban and rural settings [28], control of environmental factors and expansion of the health care system will be needed to increase physical activity of the rural elderly in the future.

Research on the relationship between carbohydrate intake and chronic disease has been reported [29,30,31]. Carbohydrates are one of the main nutrients that affect insulin secretion and postprandial blood glucose levels. When insulin secretion and blood glucose levels steadily increase, HDL-cholesterol decreases and glycated hemoglobin and oxidative stress increase, increasing the risk of chronic disease [32]. In general, the Korean diet consists of rice as a staple food, soup/stew, and side dishes which are plant- or animal-based foods. Hence, Koreans have a tendency of a high energy intake from carbohydrates and the energy contribution ratio of carbohydrate in Korean adults over the age of 19 has been reported as 61.1% [33]. Then, the energy contribution ratio of carbohydrate increases to 70.3% in the elderly aged 65 years or more [33], since the elderly are more likely to have less diverse diet with aging, leading to consumption of a smaller number of side dishes and rice only. In the 2020 Dietary Reference Intakes for Koreans (KDRI), the acceptable macronutrient distribution range (AMDR) for carbohydrates is 55 to 65% [34]. In this study, however, the energy contribution ratio of carbohydrate in the rural elderly was 73.5% for men and 77.6% for women, both significantly higher than the AMDR.

This study revealed that the energy contribution ratio of carbohydrate was significantly higher in the rural elderly (73.5% for men; 77.6% for women) than in the urban elderly (70.9% for men; 73.6% for women) and even the energy contribution ratio of protein in the rural elderly women was lower than that in the urban elderly women. These results are consistent with data from previous studies, which identified a significantly higher intake of carbohydrates in rural dwellers than in urban dwellers [4,35]. According to a study by Ha et al. [35], the odds of an energy contribution ratio of carbohydrates exceeding 65% was 1.38 times higher among the rural elderly compared to metropolitan residents. This result can be explained by the fact that rural residents are more likely to maintain a traditional Korean dietary pattern, comprising mainly rice and kimchi, than urban residents [36,37]. Previous research findings have suggested that when the energy contribution ratio of carbohydrates is higher, the intake quality of other nutrients will be lower [38,39] and the energy contribution ratio of fat and protein will be relatively lower as well. This study demonstrated that rural elderly women had a significantly lower proportion of energy intake from protein compared to their urban counterparts. Insufficient protein intake in the elderly can cause sarcopenia and frailty [40]; therefore, it is urgent that strategies are implemented to improve overall diet quality of rural elderly by increasing dietary diversity.

Although HDL-cholesterol is a predictive marker of major coronary events [41], not many studies have compared HDL-cholesterol levels between residents in urban and rural settings [42,43,44] and the studies that have been reported show inconsistent results. In a study of adults aged 25–74 years in Myanmar [45], the urban residents had a significantly higher proportion of low HDL-cholesterol, but a study of Indian adults aged 20 or older indicated no differences in urban–rural HDL-cholesterol levels [42]. Meanwhile, a study of Korean adults aged 40–70 years from the Korea Genome and Epidemiology Study dataset found that urban dwellers had a significantly higher level of HDL-cholesterol than rural dwellers [4]. In this study based on the KNHANES dataset, the odds of low HDL and high TG were significantly higher in rural elderly women than in urban elderly women after adjusting for confounding factors. These findings together suggest that differences in blood lipid levels in people from urban and rural areas may vary by country and age group, but no systematic study to investigate the difference has been reported.

A wide array of factors, including genetic and environmental factors, affect the levels of HDL-cholesterol; however, diet is a major factor related to changes in HDL-cholesterol levels [46,47]. Carbohydrates play a very important role in HDL-cholesterol levels as carbohydrate intake decreases HDL-cholesterol levels but increases TG levels. These processes can be explained from many aspects. Carbohydrates reduce HDL-cholesterol synthesis by inhibiting the transcription of lipoprotein A-I, a major component of HDL-cholesterol [48]. In addition, carbohydrates appear to accelerate the degradation of HDL-cholesterol by regulating adipokine secretion [49]. Furthermore, lipoprotein lipase (LPL) breaks down the TG in chylomicrons and VLDL, resulting in the formation of HDL remnants which may, in turn, be HDL precursors. A high carbohydrate diet inhibits LPL activity, inducing fatty acid production in hepatocytes [46], which eventually increases TG and decreases HDL-cholesterol levels. In an epidemiological study, HDL-cholesterol levels were significantly lower in the highest tertile of carbohydrate intake than in the lowest tertile of carbohydrate intake after adjusting for other confounders, such as obesity [47]. This study found that the rural elderly had higher carbohydrate intake but lower HDL-cholesterol levels than their urban counterparts, confirming previous findings.

Our sub-analysis of the association between carbohydrate intake and metabolic risks such as abdominal obesity, high TG, low HDL, and high blood glucose confirmed that the odds of low HDL and high TG were significantly higher in elderly women with high carbohydrate intake. Given high carbohydrate consumption in the rural elderly, we examined its relationship with metabolic risk (abdominal obesity, high TG, low HDL, and high blood glucose). When we compared patients with an energy contribution ratio of carbohydrate of 65% and over with those under 65%, after stratifying by sex (men/women) and region (urban/rural), there was no significant correlation between proportion of energy intake from carbohydrates and metabolic risks. In their study of adults aged 19–64 years, Soh et al. [38] reported that people with 75% or higher energy contribution ratio of carbohydrate had 1.37 times higher odds of low HDL-cholesterol compared to people with 60–65% energy contribution ratio of carbohydrate (95% CI 1.10–1.70), which was a significant difference. Several studies demonstrated that a percentage of energy from carbohydrates that was too high or too low was associated with increased mortality [50,51]. This study found no significant differences in odds ratios of metabolic indicators according to the percentage of energy intake from carbohydrates between urban and rural areas. It is difficult to explain this result, but it may be related to the low reference point (65%) of the energy contribution ratio of carbohydrates used in this study because a previous study showed a significant risk of low HDL-cholesterol only in the group with 75% or higher energy contribution ratio of carbohydrates [38]. Another possible explanation is that dividing the study population according to energy contribution ratio of carbohydrates was partial to one group when analyzing its relationship with the odds ratios of metabolic indicators after stratifying by sex and region, resulting in uneven distribution of subjects between the groups.

This study had several limitations. First, this study used cross-sectional data and, therefore, could not establish causality. Second, the dietary data used in this study were dietary information collected through a 24-h recall method, and, thus, may not fully estimate usual dietary intake. Third, in this study, data for selected variables were extracted from a large-scale national study dataset that was already established, suppressing consideration of environmental factors between urban–rural settings for analysis.

## 5. Conclusions

This study demonstrates that the rural elderly had significantly lower income and education levels and significantly higher carbohydrate intake, but lower intake of other micronutrients compared to their urban counterparts. In addition, the rural elderly women showed significantly higher ORs of high TG and low HDL-cholesterol compared to their urban counterparts. The findings of this study suggest that it is necessary to develop plans for the rural elderly women to avoid excessive carbohydrate intake and to further increase the intake of other macronutrients and micronutrients, such as protein and lipids. Moreover, the proportion of low HDL-cholesterol was higher in elderly women than in elderly men and this pattern was particularly pronounced in the elderly women in rural areas. As low HDL-cholesterol is a major predictor of cardiovascular disease, further research is required to identify the main factors, especially dietary factors, that affect low HDL-cholesterol levels in rural elderly in a systematic and detailed manner.

## Figures and Tables

**Table 1:** Socio-demographic factors of subjects.

		Men (*n* = 1358)			Women (*n* = 1660)		
		Urban	Rural	*p*-Value	Urban	Rural	*p*-Value
N		995	363		1241	419	
Age (years)		71.7 ± 0.2	72.3 ± 0.3	0.049	71.9 ± 0.2	72.8 ± 0.3	0.010
	65–74	676 (69.6)	232 (64.1)	0.077	854 (68.6)	251 (60.0)	0.008
	≥75	319 (30.4)	131 (35.9)		387 (31.4)	168 (40.0)	
Education level (%)	≤Elementary	374 (37.5)	176 (48.3)	<0.0001	835 (66.0)	360 (85.1)	<0.0001
	≤Middle school	169 (17.2)	77 (21.6)		166 (12.9)	29 (7.5)	
	≥High school	452 (45.3)	110 (30.0)		240 (21.1)	30 (7.4)	
Household income (%)	Low	360 (35.6)	184 (52.1)	<0.0001	594 (47.0)	260 (62.6)	<0.0001
	Middle-low	303 (29.2)	109 (30.0)		320 (24.5)	96 (22.0)	
	Middle-high	183 (19.7)	51 (14.3)		185 (16.3)	38 (9.6)	
	High	144 (15.4)	17 (3.6)		134 (12.2)	22 (5.8)	
Marital status (%)	Married	865 (87.3)	325 (90.4)	0.163	645 (50.3)	222 (50.1)	0.966
	Others ^(1)^	130 (12.7)	38 (9.6)		596 (49.7)	197 (49.9)	
Alcohol drinking (%)	No	397 (39.7)	149 (41.7)	0.546	1000 (80.6)	353 (83.2)	0.321
	Yes	598 (60.3)	214 (58.3)		241 (9.4)	66 (16.8)	
Smoking status (%)	Past/never	801 (81.0)	302 (83.2)	0.380	1214 (97.9)	410 (97.1)	0.461
	Current	194 (19.0)	61 (16.8)		27 (2.1)	9 (2.9)	
Physical activity	No	405 (52.1)	183 (60.5)	0.040	625 (66.7)	239 (72.5)	0.087
	Yes	358 (47.9)	105 (39.5)		303 (33.6)	78 (27.5)	
BMI (kg/m^2^)		23.8 ± 0.1	23.9 ± 0.2	0.645	24.8 ± 0.1	24.4 ± 0.2	0.157
Obesity degree ^(2)^	Underweight	32 (3.2)	9 (2.8)	0.137	16 (1.5)	16 (4.4)	0.043
	Normal	359 (35.2)	138 (38.1)		380 (29.0)	131 (29.5)	
	Overweight	287 (30.1)	80 (22.4)		307 (24.9)	96 (22.3)	
	Obesity	317 (31.5)	136 (36.7)		538 (44.7)	176 (43.7)	

Data represent mean ± standard error or number of case (%). ^(1)^ Widowed, separated, divorced, or never married. ^(2)^ underweight: BMI < 18.5 kg/m^2^; normal weight: 18.5 kg/m^2^ ≤ BMI < 23 kg/m^2^; overweight: 23 kg/m^2^ ≤ BMI < 25 kg/m^2^; or obesity: BMI ≥ 25 kg/m^2^.

**Table 2:** Status of the metabolic abnormalities indicators of participants.

	Men (*n* = 1358)			Women (*n* = 1660)		
	Urban	Rural	*p*-Value	Urban	Rural	*p*-Value
N	995	363		1241	419	
Waist circumference (cm)	86.3 ± 0.3	86.5 ± 0.5	0.752	84.6 ± 0.4	84.1 ± 0.6	0.562
TG (mg/dL)	131.6 ± 3.0	142.2 ± 5.0	0.069	133.4 ± 2.2	150.0 ± 6.3	0.014
HDL-cholesterol (mg/dL)	46.2 ± 0.4	45.3 ± 0.6	0.232	50.0 ± 0.4	48.1 ± 0.6	0.005
Fasting blood glucose (mg/dL)	107.2 ± 0.9	106.3 ± 1.5	0.623	105.7 ± 0.8	103.7 ± 1.5	0.254
Prevalence of metabolic abnormalities (%) ^(1)^						
Abdominal obesity	321 (32.2)	133 (35.5)	0.306	562 (46.5)	192 (46.6)	0.982
High TG	303 (30.2)	123 (34.1)	0.228	393 (29.5)	167 (39.5)	0.002
Low HDL-cholesterol	297 (31.3)	129 (35.6)	0.157	684 (54.1)	259 (61.9)	0.017
High blood glucose	568 (55.4)	197 (52.5)	0.393	620 (50.2)	183 (44.5)	0.126

Data represent mean ± standard error or number of case (%). ^(1)^ Abdominal obesity: a waist circumference ≥ 90 cm in men and ≥80 cm in women; High TG: ≥150 mg/dL; Low HDL-cholesterol: <40 mg/dL in men and <50 mg/dL in women; Hyper blood glucose: ≥100 mg/dL or medication (insulin or oral agents).

**Table 3:** Energy and nutrient intakes of participants.

	Men (*n* = 1358)			Women (*n* = 1660)		
	Urban	Rural	*p*-Value	Urban	Rural	*p*-Value
N	995	363		1241	419	
Energy (kcal)	1958.4 ± 25.6	2033.1 ± 44.8	0.149	1475.4 ± 17.8	1527.5 ± 31.1	0.143
Carbohydrate (g)	326.9 ± 4.4	347.5 ± 6.9	0.012	266.2 ± 3.2	290.8 ± 6.4	0.001
Protein (g)	64.7 ± 1.2	64.7 ± 2.1	0.992	47.3 ± 0.8	45.5 ± 1.4	0.253
Fat (g)	32.7 ± 0.9	28.8 ± 1.4	0.019	23.1 ± 0.6	18.0 ± 1.0	<0.0001
Fiber (g)	26.6 ± 0.5	25.2 ± 1.1	0.251	22.0 ± 0.4	20.4 ± 0.8	0.087
Cholesterol (mg)	178.8 ± 7.1	155.9 ± 11.3	0.087	126.1 ± 5.1	95.0 ± 8.7	0.002
Calcium (mg)	491.2 ± 14.4	442.5 ± 17.3	0.031	372.4 ± 7.8	352.7 ± 16.9	0.292
Phosphorus (mg)	1045.6 ± 17.8	1018.5 ± 33.9	0.481	791.2 ± 11.9	749.4 ± 22.9	0.106
Iron (mg)	18.1 ± 0.4	18.2 ± 0.6	0.964	15.0 ± 0.6	14.0 ± 0.5	0.255
Sodium (mg)	3909.2 ± 90.4	3905.2 ± 140.4	0.565	2640.0 ± 59.4	2744.3 ± 119.0	0.434
Potassium (mg)	3104.0 ± 59.0	2980.8 ± 97.9	0.280	2491.0 ± 45.6	2417.2 ± 93.3	0.476
Vitamin A (ug RE)	736.1 ± 32.8	618.1 ± 45.5	0.037	582.9 ± 23.3	488.7 ± 41.6	0.048
Vitamin B_1_ (mg)	2.0 ± 0.03	2.0 ± 0.06	0.404	1.5 ± 0.02	1.5 ± 0.04	0.693
Vitamin B_2_ (mg)	1.2 ± 0.03	1.1 ± 0.04	0.011	0.9 ± 0.02	0.8 ± 0.04	0.007
Niacin (mg)	15.3 ± 0.3	14.7 ± 0.5	0.347	11.0 ± 0.2	10.3 ± 0.3	0.111
Vitamin C (mg)	115.8 ± 4.5	97.6 ± 7.0	0.029	104.6 ± 3.9	79.9 ± 6.3	0.001
% E of carbohydrate	70.9 ± 0.3	73.5 ± 0.6	0.001	73.5 ± 0.3	77.6 ± 0.6	<0.0001
% E of protein	13.9 ± 0.2	13.5 ± 0.3	0.209	12.8 ± 0.1	12.0 ± 0.2	0.001
% E of fat	15.2 ± 0.3	13.0 ± 0.5	<0.0001	13.7 ± 0.3	10.4 ± 0.4	<0.0001

Data represent mean ± standard error.

**Table 4:** OR of metabolic abnormalities of rural and urban elderly.

	Men (*n* = 1358)			Women (*n* = 1660)		
	Urban	Rural	*p*-Value	Urban	Rural	*p*-Value
N	995	363		1241	419	
Abdominal obesity						
Model 1 ^(2)^	1	1.16 (0.87–1.55) ^(1)^	0.308	1	1.00 (0.76–1.33)	0.982
Model 2 ^(3)^	1	1.18 (0.83–1.69)	0.914	1	0.99 (0.64–1.52)	0.945
High TG						
Model 1	1	1.20 (0.89–1.61)	0.229	1	1.56 (1.17–2.09)	0.003
Model 2	1	1.24 (0.88–1.74)	0.215	1	1.66 (1.23–2.23)	<0.001
Low HDL-cholesterol						
Model 1	1	1.21 (0.93–1.58)	0.156	1	1.38 (1.06–1.80)	0.017
Model 2	1	0.86 (0.63–1.18)	0.361	1	1.33 (1.01–1.77)	0.046
High blood glucose						
Model 1	1	0.89 (0.68–1.17)	0.394	1	0.80 (0.59–1.07)	0.128
Model 2	1	0.76 (0.55–1.05)	0.092	1	0.84 (0.62–1.14)	0.269

^(1)^ Values are expressed as odds ratios (confidence intervals). ^(2)^ Model 1: Crude. ^(3)^ Model 2: Adjusted for age, education level, household income, physical activity, and total energy intake in men; adjusted for age, education level, household income, obesity degree, and total energy intake in women.

**Table 5:** OR of metabolic abnormalities according to quartile of carbohydrate consumption ^(1)^.

	Men					Women				
	Q1	Q2	Q3	Q4	*p* for Trend	Q1	Q2	Q3	Q4	*p* for Trend
N	339	340	340	339		415	415	415	415	
Median (g)	211.08	285.88	356.11	463.33		169.69	230.34	290.86	389.22	
Abdominal obesity										
Model 1 ^(2)^	1	1.20 (0.85–1.69)	0.84 (0.59–1.20)	1.15 (0.80–1.66)	0.802	1	0.91 (0.67–1.25)	0.94 (0.68–1.29)	1.14 (0.84–1.56)	0.336
Model 2 ^(3)^	1	1.09 (0.73–1.63)	0.78 (0.49–1.24)	1.15 (0.62–2.15)	0.875	1	1.12 (0.70–1.80)	0.93 (0.54–1.61)	1.32 (0.60–2.90)	0.617
High TG										
Model 1	1	0.65 (0.46–0.92)	0.68 (0.47–0.98)	0.69 (0.48–0.99)	0.084	1	0.78 (0.57–1.08)	1.06 (0.77–1.46)	1.07 (0.77–1.48)	0.340
Model 2	1	0.58 (0.39–0.88)	0.83 (0.51–1.33)	0.68 (0.37–1.27)	0.426	1	0.94 (0.66–1.36)	1.42 (0.92–2.21)	1.84 (0.95–3.54)	0.036
Low HDL-cholesterol										
Model 1	1	0.99 (0.69–1.42)	0.95 (0.66–1.37)	0.94 (0.66–1.33)	0.688	1	1.01 (0.73–1.38)	1.74 (1.26–2.39)	0.94 (0.69–1.29)	0.796
Model 2	1	1.04 (0.67–1.64)	1.36 (0.81–2.28)	1.72 (0.89–3.32)	0.087	1	1.31 (0.93–1.85)	2.42 (1.59–3.68)	1.67 (0.91–3.05)	0.040
High blood glucose										
Model 1	1	1.12 (0.80–1.58)	0.89 (0.63–1.25)	0.71 (0.51–0.98)	0.014	1	0.67 (0.49–0.92)	0.74 (0.54–1.01)	0.75 (0.54–1.03)	0.187
Model 2	1	1.10 (0.72–1.67)	0.89 (0.56–1.43)	0.65 (0.38–1.12)	0.080	1	0.69 (0.48–0.98)	0.71 (0.47–1.07)	0.70 (0.38–1.28)	0.330

^(1)^ Values are expressed as odds ratios (confidence intervals). ^(2)^ Model 1: Crude. ^(3)^ Model 2: Adjusted for age, education level, household income, physical activity, living area, and total energy intake in men; Adjusted for age, education, household income, obesity degree, living area, and total energy intake in women.

**Table 6:** OR of metabolic abnormalities according to the percentage of energy from carbohydrate in men ^(1)^.

	Urban			Rural		
	≤65% of Carbohydrate (% E)	>65% of Carbohydrate (% E)	*p* Value	≤65% of Carbohydrate (% E)	>65% of Carbohydrate (% E)	*p* Value
N	251	744		62	301	
Median (%)	59.10	75.25		60.00	77.07	
Abdominal obesity						
Model 1 ^(2)^	1	0.79 (0.55–1.11)	0.175	1	1.01 (0.54–1.88)	0.976
Model 2 ^(3)^	1	0.90 (0.59–1.36)	0.607	1	1.50 (0.74–3.05)	0.263
High TG						
Model 1	1	1.10 (0.76–1.58)	0.622	1	1.49 (0.77–2.89)	0.241
Model 2	1	1.07 (0.71–1.60)	0.743	1	1.06 (0.51–2.20)	0.866
Low HDL-cholesterol						
Model 1	1	1.25 (0.88–1.78)	0.222	1	1.94 (1.06–3.56)	0.033
Model 2	1	0.95 (0.63–1.44)	0.816	1	1.78 (0.87–3.61)	0.112
High blood glucose						
Model 1	1	0.85 (0.62–1.16)	0.304	1	1.32 (0.78–2.23)	0.299
Model 2	1	0.81 (0.55–1.17)	0.256	1	1.54 (0.91–2.62)	0.108

^(1)^ Values are expressed as odds ratios (confidence intervals). ^(2)^ Model 1: Crude. ^(3)^ Model 2: Adjusted for age, education level, household income, and total energy intake.

**Table 7:** OR of metabolic abnormalities according to the percentage of energy from carbohydrate in women ^(1)^.

	Urban			Rural		
	≤65% of Carbohydrate (% E)	>65% of Carbohydrate (% E)	*p*-Value	≤65% of Carbohydrate (% E)	>65% of Carbohydrate (% E)	*p*-Value
N	222	1019		41	378	
Median (%)	59.70	77.09		60.83	79.84	
Abdominal obesity						
Model 1 ^(2)^	1	0.80 (0.56–1.15)	0.230	1	1.53 (0.76–3.05)	0.232
Model 2 ^(3)^	1	0.88 (0.55–1.41)	0.589	1	1.22 (0.50–2.96)	0.661
High TG						
Model 1	1	1.18 (0.82–1.70)	0.369	1	0.97 (0.42–2.20)	0.933
Model 2	1	1.25 (0.85–1.83)	0.263	1	0.95 (0.42–2.18)	0.905
Low HDL-cholesterol						
Model 1	1	1.35 (0.98–1.86)	0.068	1	0.80 (0.37–1.78)	0.589
Model 2	1	1.35 (0.94–1.93)	0.103	1	0.82 (0.35–1.93)	0.649
High blood glucose						
Model 1	1	1.12 (0.81–1.56)	0.500	1	0.85 (0.42–1.75)	0.663
Model 2	1	1.08 (0.78–1.51)	0.640	1	0.92 (0.43–1.97)	0.834

^(1)^ Values are expressed as odds ratios (confidence intervals). ^(2)^ Model 1: Crude. ^(3)^ Model 2: Adjusted for age, education level, household income, and total energy intake.

## Data Availability

The KNHANES data used in the manuscript can be found at the following link: https://knhanes.kdca.go.kr/knhanes/main.do (accessed on 10 February 2021).
